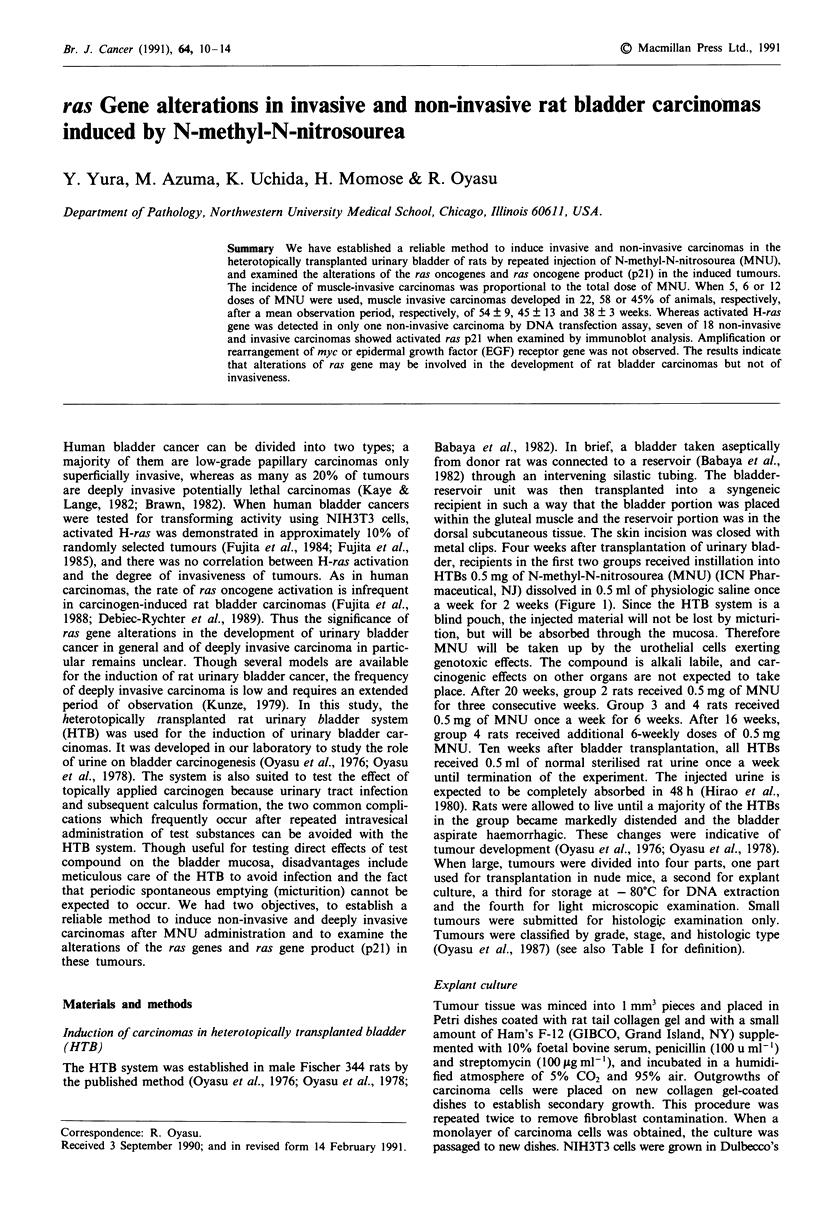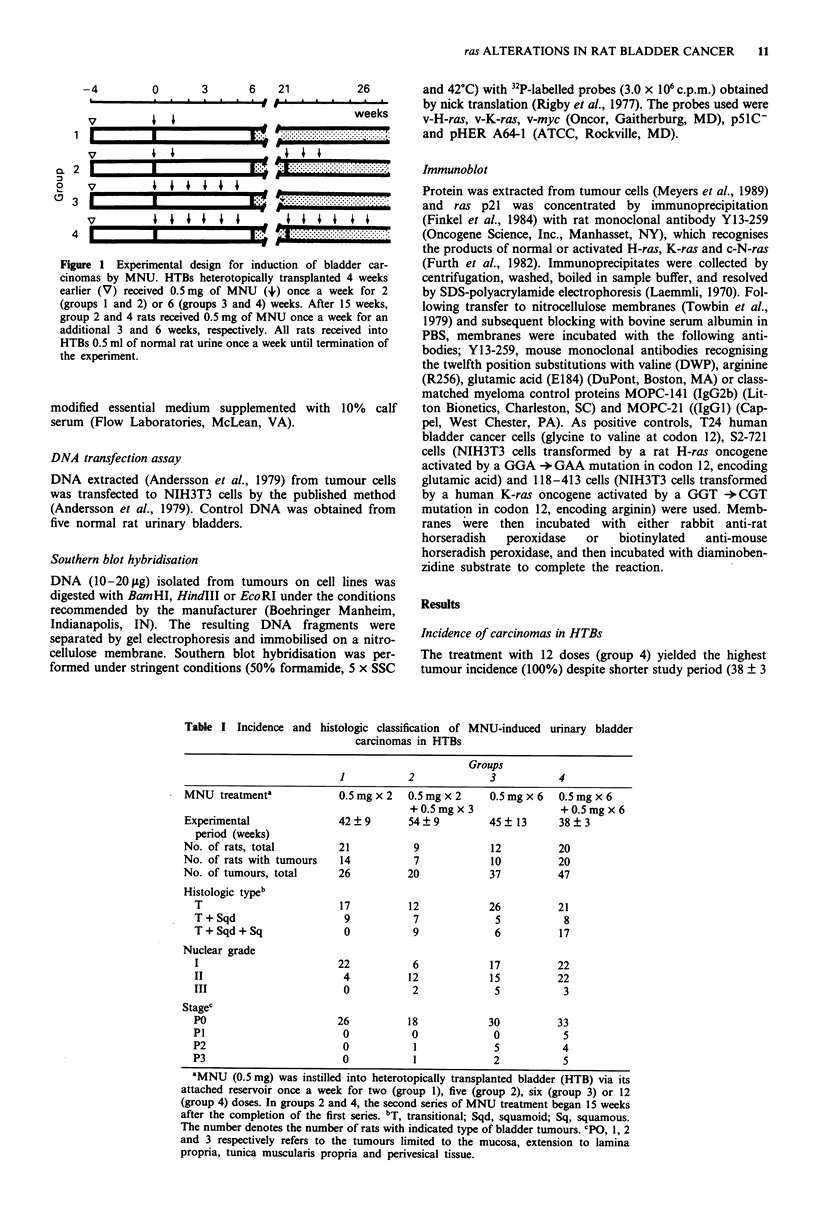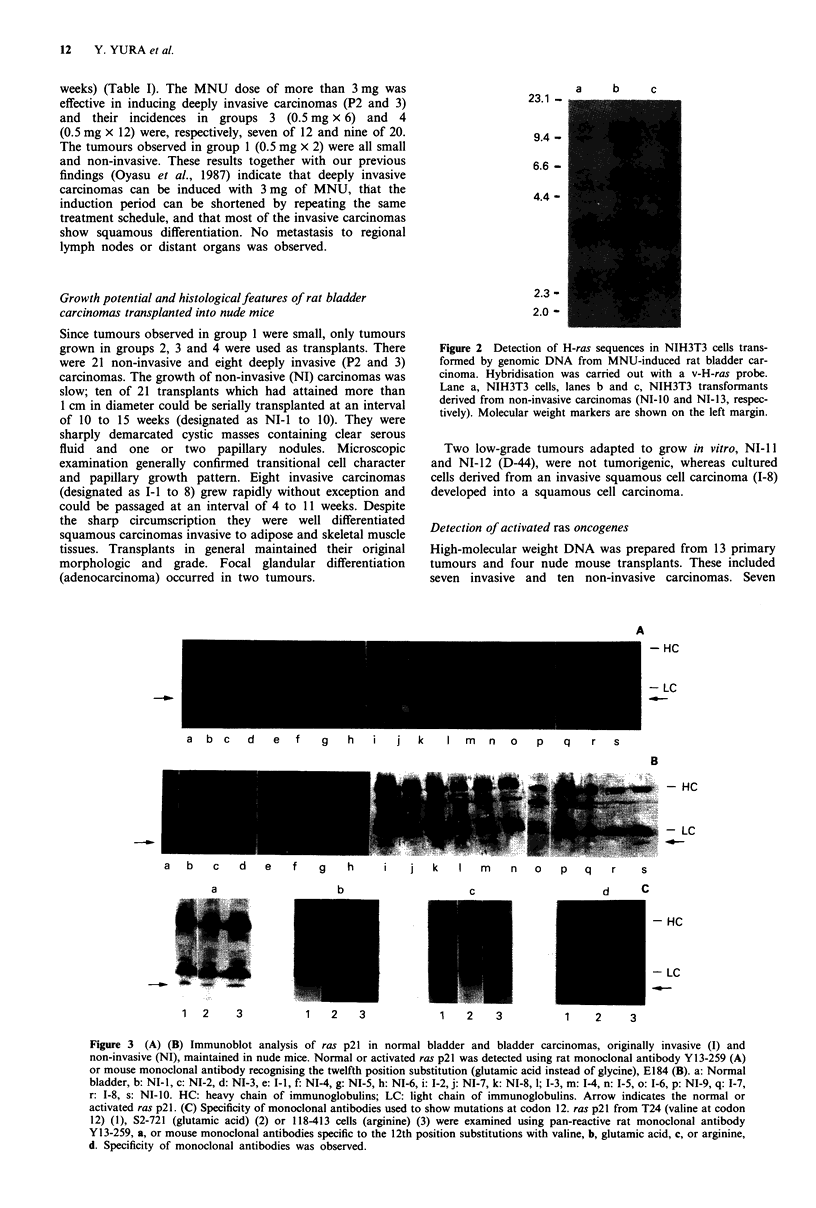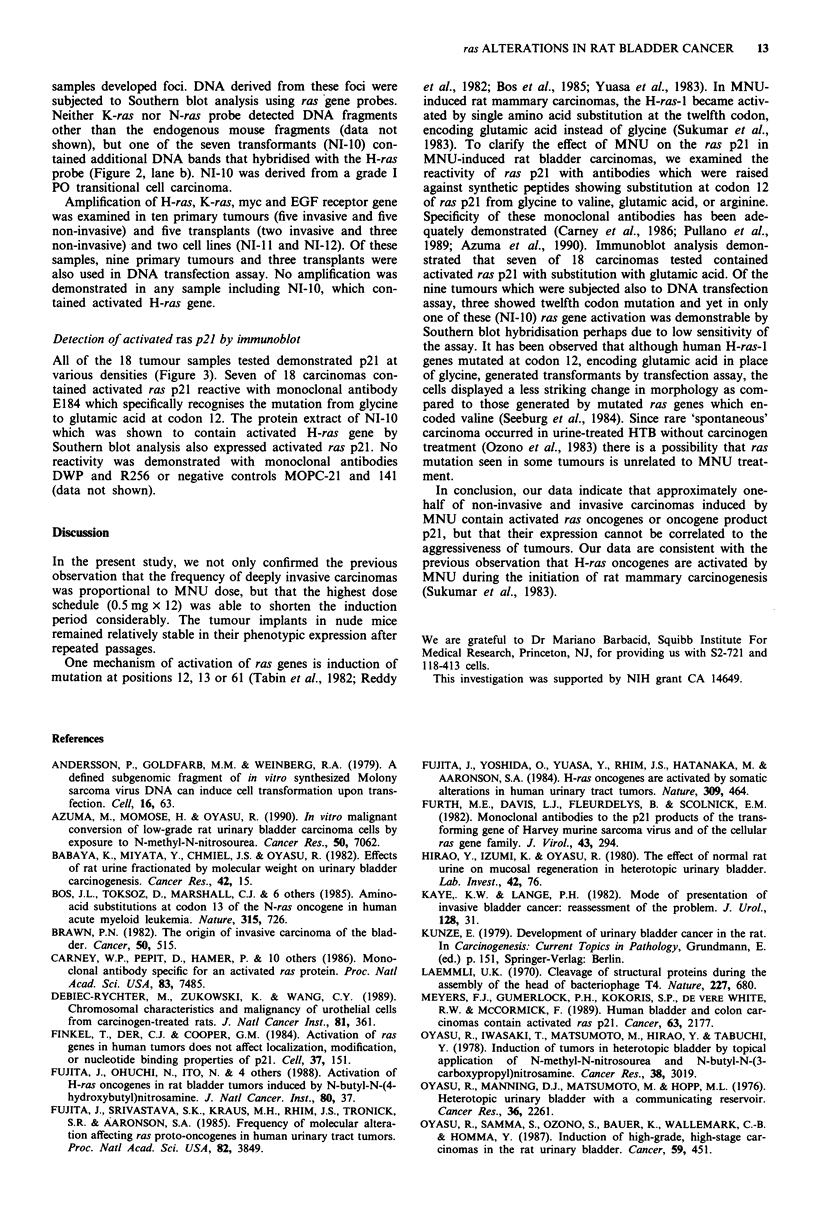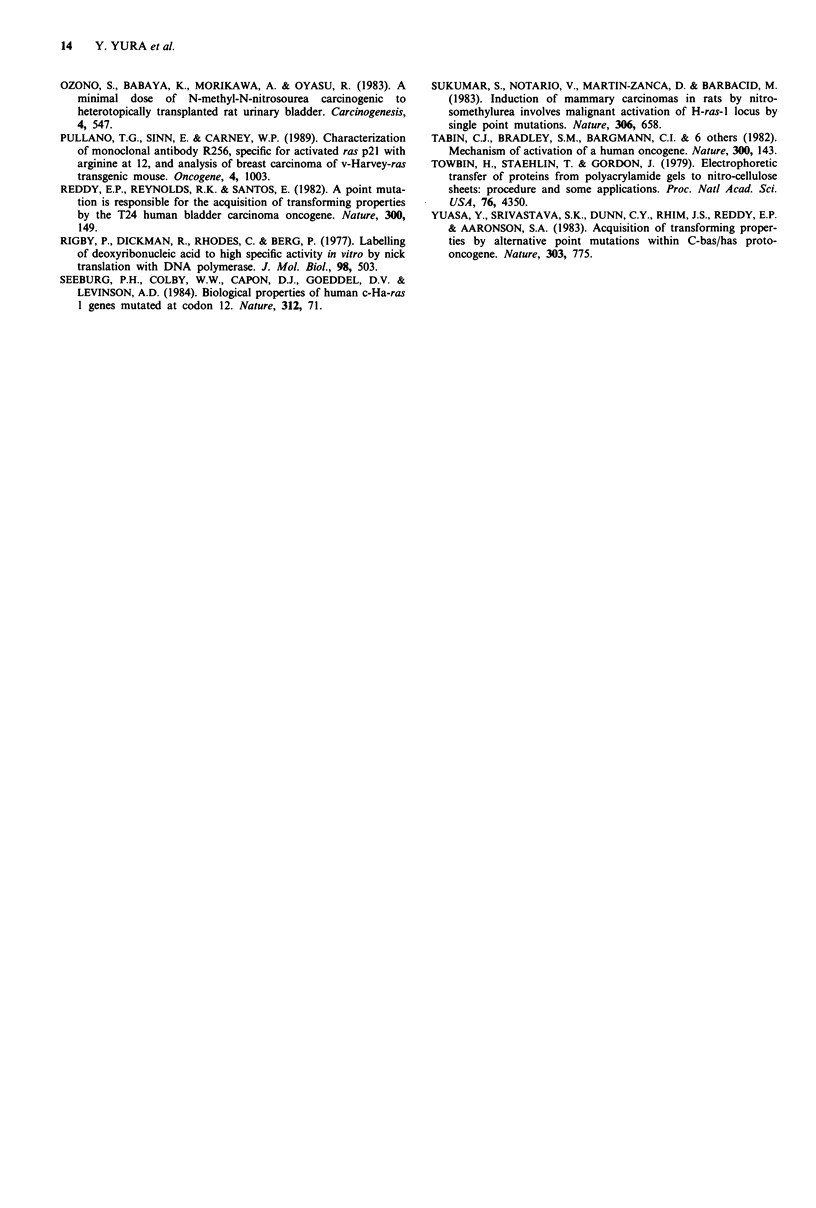# ras gene alterations in invasive and non-invasive rat bladder carcinomas induced by N-methyl-N-nitrosourea.

**DOI:** 10.1038/bjc.1991.231

**Published:** 1991-07

**Authors:** Y. Yura, M. Azuma, K. Uchida, H. Momose, R. Oyasu

**Affiliations:** Department of Pathology, Northwestern University Medical School, Chicago, Illinois 60611.

## Abstract

**Images:**


Br. J. Cancer (1991), 64, 10-14 © Macmillan Press Ltd., 1991

### *ras* Gene alterations in invasive and non-invasive rat bladder carcinomas induced by N-methyl-N-nitrosourea

Y. Yura, M. Azuma, K. Uchida, H. Momose & R. Oyasu

*Department of Pathology, Northwestern University Medical School, Chicago, Illinois 60611, USA.*

**Summary** We have established a reliable method to induce invasive and non-invasive carcinomas in the heterotopically transplanted urinary bladder of rats by repeated injection of N-methyl-N-nitrosourea (MNU), and examined the alterations of the *ras* oncogenes and *ras* oncogene product (p21) in the induced tumours. The incidence of muscle-invasive carcinomas was proportional to the total dose of MNU. When 5, 6 or 12 doses of MNU were used, muscle invasive carcinomas developed in 22, 58 or 45% of animals, respectively, after a mean observation period, respectively, of $54 \pm 9$, $45 \pm 13$ and $38 \pm 3$ weeks. Whereas activated H-*ras* gene was detected in only one non-invasive carcinoma by DNA transfection assay, seven of 18 non-invasive and invasive carcinomas showed activated *ras* p21 when examined by immunoblot analysis. Amplification or rearrangement of *myc* or epidermal growth factor (EGF) receptor gene was not observed. The results indicate that alterations of *ras* gene may be involved in the development of rat bladder carcinomas but not of invasiveness.

Human bladder cancer can be divided into two types; a majority of them are low-grade papillary carcinomas only superficially invasive, whereas as many as 20% of tumours are deeply invasive potentially lethal carcinomas (Kaye & Lange, 1982; Brawn, 1982). When human bladder cancers were tested for transforming activity using NIH3T3 cells, activated H-*ras* was demonstrated in approximately 10% of randomly selected tumours (Fujita *et al.*, 1984; Fujita *et al.*, 1985), and there was no correlation between H-*ras* activation and the degree of invasiveness of tumours. As in human carcinomas, the rate of *ras* oncogene activation is infrequent in carcinogen-induced rat bladder carcinomas (Fujita *et al.*, 1988; Debiec-Rychter *et al.*, 1989). Thus the significance of *ras* gene alterations in the development of urinary bladder cancer in general and of deeply invasive carcinoma in particular remains unclear. Though several models are available for the induction of rat urinary bladder cancer, the frequency of deeply invasive carcinoma is low and requires an extended period of observation (Kunze, 1979). In this study, the *h*eterotopically *t*ransplanted rat urinary *b*ladder system (HTB) was used for the induction of urinary bladder carcinomas. It was developed in our laboratory to study the role of urine on bladder carcinogenesis (Oyasu *et al.*, 1976; Oyasu *et al.*, 1978). The system is also suited to test the effect of topically applied carcinogen because urinary tract infection and subsequent calculus formation, the two common complications which frequently occur after repeated intravesical administration of test substances can be avoided with the HTB system. Though useful for testing direct effects of test compound on the bladder mucosa, disadvantages include meticulous care of the HTB to avoid infection and the fact that periodic spontaneous emptying (micturition) cannot be expected to occur. We had two objectives, to establish a reliable method to induce non-invasive and deeply invasive carcinomas after MNU administration and to examine the alterations of the *ras* genes and *ras* gene product (p21) in these tumours.

### Materials and methods

### *Induction of carcinomas in heterotopically transplanted bladder (HTB)*

The HTB system was established in male Fischer 344 rats by the published method (Oyasu *et al.*, 1976; Oyasu *et al.*, 1978; Babaya *et al.*, 1982). In brief, a bladder taken aseptically from donor rat was connected to a reservoir (Babaya *et al.*, 1982) through an intervening silastic tubing. The bladder-reservoir unit was then transplanted into a syngeneic recipient in such a way that the bladder portion was placed within the gluteal muscle and the reservoir portion was in the dorsal subcutaneous tissue. The skin incision was closed with metal clips. Four weeks after transplantation of urinary bladder, recipients in the first two groups received instillation into HTBs 0.5 mg of N-methyl-N-nitrosourea (MNU) (ICN Pharmaceutical, NJ) dissolved in 0.5 ml of physiologic saline once a week for 2 weeks (Figure 1). Since the HTB system is a blind pouch, the injected material will not be lost by micturition, but will be absorbed through the mucosa. Therefore MNU will be taken up by the urothelial cells exerting genotoxic effects. The compound is alkali labile, and carcinogenic effects on other organs are not expected to take place. After 20 weeks, group 2 rats received 0.5 mg of MNU for three consecutive weeks. Group 3 and 4 rats received 0.5 mg of MNU once a week for 6 weeks. After 16 weeks, group 4 rats received additional 6-weekly doses of 0.5 mg MNU. Ten weeks after bladder transplantation, all HTBs received 0.5 ml of normal sterilised rat urine once a week until termination of the experiment. The injected urine is expected to be completely absorbed in 48 h (Hirao *et al.*, 1980). Rats were allowed to live until a majority of the HTBs in the group became markedly distended and the bladder aspirate haemorrhagic. These changes were indicative of tumour development (Oyasu *et al.*, 1976; Oyasu *et al.*, 1978). When large, tumours were divided into four parts, one part used for transplantation in nude mice, a second for explant culture, a third for storage at $-80°C$ for DNA extraction and the fourth for light microscopic examination. Small tumours were submitted for histologic examination only. Tumours were classified by grade, stage, and histologic type (Oyasu *et al.*, 1987) (see also Table I for definition).

### *Explant culture*

Tumour tissue was minced into 1 $mm^3$ pieces and placed in Petri dishes coated with rat tail collagen gel and with a small amount of Ham's F-12 (GIBCO, Grand Island, NY) supplemented with 10% foetal bovine serum, penicillin (100 u $ml^{-1}$) and streptomycin (100 $\mu g\ ml^{-1}$), and incubated in a humidified atmosphere of 5% $CO_2$ and 95% air. Outgrowths of carcinoma cells were placed on new collagen gel-coated dishes to establish secondary growth. This procedure was repeated twice to remove fibroblast contamination. When a monolayer of carcinoma cells was obtained, the culture was passaged to new dishes. NIH3T3 cells were grown in Dulbecco's

Correspondence: R. Oyasu.
Received 3 September 1990; and in revised form 14 February 1991.

**Figure 1** Experimental design for induction of bladder carcinomas by MNU. HTBs heterotopically transplanted 4 weeks earlier (▽) received 0.5 mg of MNU (↓) once a week for 2 (groups 1 and 2) or 6 (groups 3 and 4) weeks. After 15 weeks, group 2 and 4 rats received 0.5 mg of MNU once a week for an additional 3 and 6 weeks, respectively. All rats received into HTBs 0.5 ml of normal rat urine once a week until termination of the experiment.

modified essential medium supplemented with 10% calf serum (Flow Laboratories, McLean, VA).

*DNA transfection assay*

DNA extracted (Andersson *et al.*, 1979) from tumour cells was transfected to NIH3T3 cells by the published method (Andersson *et al.*, 1979). Control DNA was obtained from five normal rat urinary bladders.

*Southern blot hybridisation*

DNA (10–20 μg) isolated from tumours on cell lines was digested with *Bam*HI, *Hind*III or *Eco*RI under the conditions recommended by the manufacturer (Boehringer Manheim, Indianapolis, IN). The resulting DNA fragments were separated by gel electrophoresis and immobilised on a nitrocellulose membrane. Southern blot hybridisation was performed under stringent conditions (50% formamide, 5 × SSC and 42°C) with $^{32}$P-labelled probes (3.0 × $10^6$ c.p.m.) obtained by nick translation (Rigby *et al.*, 1977). The probes used were v-H-*ras*, v-K-*ras*, v-*myc* (Oncor, Gaitherburg, MD), p51C$^-$ and pHER A64-1 (ATCC, Rockville, MD).

*Immunoblot*

Protein was extracted from tumour cells (Meyers *et al.*, 1989) and *ras* p21 was concentrated by immunoprecipitation (Finkel *et al.*, 1984) with rat monoclonal antibody Y13-259 (Oncogene Science, Inc., Manhasset, NY), which recognises the products of normal or activated H-*ras*, K-*ras* and c-N-*ras* (Furth *et al.*, 1982). Immunoprecipitates were collected by centrifugation, washed, boiled in sample buffer, and resolved by SDS-polyacrylamide electrophoresis (Laemmli, 1970). Following transfer to nitrocellulose membranes (Towbin *et al.*, 1979) and subsequent blocking with bovine serum albumin in PBS, membranes were incubated with the following antibodies; Y13-259, mouse monoclonal antibodies recognising the twelfth position substitutions with valine (DWP), arginine (R256), glutamic acid (E184) (DuPont, Boston, MA) or class-matched myeloma control proteins MOPC-141 (IgG2b) (Litton Bionetics, Charleston, SC) and MOPC-21 ((IgG1) (Cappel, West Chester, PA). As positive controls, T24 human bladder cancer cells (glycine to valine at codon 12), S2-721 cells (NIH3T3 cells transformed by a rat H-*ras* oncogene activated by a GGA → GAA mutation in codon 12, encoding glutamic acid) and 118–413 cells (NIH3T3 cells transformed by a human K-*ras* oncogene activated by a GGT → CGT mutation in codon 12, encoding arginin) were used. Membranes were then incubated with either rabbit anti-rat horseradish peroxidase or biotinylated anti-mouse horseradish peroxidase, and then incubated with diaminobenzidine substrate to complete the reaction.

### Results

*Incidence of carcinomas in HTBs*

The treatment with 12 doses (group 4) yielded the highest tumour incidence (100%) despite shorter study period (38 ± 3

**Table I** Incidence and histologic classification of MNU-induced urinary bladder carcinomas in HTBs

| | *Groups* 1 | 2 | 3 | 4 |
|---|---|---|---|---|
| MNU treatment[a] | 0.5 mg × 2 | 0.5 mg × 2 + 0.5 mg × 3 | 0.5 mg × 6 | 0.5 mg × 6 + 0.5 mg × 6 |
| Experimental period (weeks) | 42 ± 9 | 54 ± 9 | 45 ± 13 | 38 ± 3 |
| No. of rats, total | 21 | 9 | 12 | 20 |
| No. of rats with tumours | 14 | 7 | 10 | 20 |
| No. of tumours, total | 26 | 20 | 37 | 47 |
| Histologic type[b] | | | | |
| T | 17 | 12 | 26 | 21 |
| T + Sqd | 9 | 7 | 5 | 8 |
| T + Sqd + Sq | 0 | 9 | 6 | 17 |
| Nuclear grade | | | | |
| I | 22 | 6 | 17 | 22 |
| II | 4 | 12 | 15 | 22 |
| III | 0 | 2 | 5 | 3 |
| Stage[c] | | | | |
| P0 | 26 | 18 | 30 | 33 |
| P1 | 0 | 0 | 0 | 5 |
| P2 | 0 | 1 | 5 | 4 |
| P3 | 0 | 1 | 2 | 5 |

[a]MNU (0.5 mg) was instilled into heterotopically transplanted bladder (HTB) via its attached reservoir once a week for two (group 1), five (group 2), six (group 3) or 12 (group 4) doses. In groups 2 and 4, the second series of MNU treatment began 15 weeks after the completion of the first series. [b]T, transitional; Sqd, squamoid; Sq, squamous. The number denotes the number of rats with indicated type of bladder tumours. [c]PO, 1, 2 and 3 respectively refers to the tumours limited to the mucosa, extension to lamina propria, tunica muscularis propria and perivesical tissue.

weeks) (Table I). The MNU dose of more than 3 mg was effective in inducing deeply invasive carcinomas (P2 and 3) and their incidences in groups 3 (0.5 mg × 6) and 4 (0.5 mg × 12) were, respectively, seven of 12 and nine of 20. The tumours observed in group 1 (0.5 mg × 2) were all small and non-invasive. These results together with our previous findings (Oyasu et al., 1987) indicate that deeply invasive carcinomas can be induced with 3 mg of MNU, that the induction period can be shortened by repeating the same treatment schedule, and that most of the invasive carcinomas show squamous differentiation. No metastasis to regional lymph nodes or distant organs was observed.

*Growth potential and histological features of rat bladder carcinomas transplanted into nude mice*

Since tumours observed in group 1 were small, only tumours grown in groups 2, 3 and 4 were used as transplants. There were 21 non-invasive and eight deeply invasive (P2 and 3) carcinomas. The growth of non-invasive (NI) carcinomas was slow; ten of 21 transplants which had attained more than 1 cm in diameter could be serially transplanted at an interval of 10 to 15 weeks (designated as NI-1 to 10). They were sharply demarcated cystic masses containing clear serous fluid and one or two papillary nodules. Microscopic examination generally confirmed transitional cell character and papillary growth pattern. Eight invasive carcinomas (designated as I-1 to 8) grew rapidly without exception and could be passaged at an interval of 4 to 11 weeks. Despite the sharp circumscription they were well differentiated squamous carcinomas invasive to adipose and skeletal muscle tissues. Transplants in general maintained their original morphologic and grade. Focal glandular differentiation (adenocarcinoma) occurred in two tumours.

**Figure 2** Detection of H-*ras* sequences in NIH3T3 cells transformed by genomic DNA from MNU-induced rat bladder carcinoma. Hybridisation was carried out with a v-H-*ras* probe. Lane a, NIH3T3 cells, lanes b and c, NIH3T3 transformants derived from non-invasive carcinomas (NI-10 and NI-13, respectively). Molecular weight markers are shown on the left margin.

Two low-grade tumours adapted to grow *in vitro*, NI-11 and NI-12 (D-44), were not tumorigenic, whereas cultured cells derived from an invasive squamous cell carcinoma (I-8) developed into a squamous cell carcinoma.

*Detection of activated* ras *oncogenes*

High-molecular weight DNA was prepared from 13 primary tumours and four nude mouse transplants. These included seven invasive and ten non-invasive carcinomas. Seven

**Figure 3** (**A**) (**B**) Immunoblot analysis of *ras* p21 in normal bladder and bladder carcinomas, originally invasive (I) and non-invasive (NI), maintained in nude mice. Normal or activated *ras* p21 was detected using rat monoclonal antibody Y13-259 (**A**) or mouse monoclonal antibody recognising the twelfth position substitution (glutamic acid instead of glycine), E184 (**B**). a: Normal bladder, b: NI-1, c: NI-2, d: NI-3, e: I-1, f: NI-4, g: NI-5, h: NI-6, i: I-2, j: NI-7, k: NI-8, l; I-3, m: I-4, n: I-5, o: I-6, p: NI-9, q: I-7, r: I-8, s: NI-10. HC: heavy chain of immunoglobulins; LC: light chain of immunoglobulins. Arrow indicates the normal or activated *ras* p21. (**C**) Specificity of monoclonal antibodies used to show mutations at codon 12. *ras* p21 from T24 (valine at codon 12) (1), S2-721 (glutamic acid) (2) or 118-413 cells (arginine) (3) were examined using pan-reactive rat monoclonal antibody Y13-259, **a**, or mouse monoclonal antibodies specific to the 12th position substitutions with valine, **b**, glutamic acid, **c**, or arginine, **d**. Specificity of monoclonal antibodies was observed.

samples developed foci. DNA derived from these foci were subjected to Southern blot analysis using *ras* gene probes. Neither K-*ras* nor N-*ras* probe detected DNA fragments other than the endogenous mouse fragments (data not shown), but one of the seven transformants (NI-10) contained additional DNA bands that hybridised with the H-*ras* probe (Figure 2, lane b). NI-10 was derived from a grade I PO transitional cell carcinoma.

Amplification of H-*ras*, K-*ras*, myc and EGF receptor gene was examined in ten primary tumours (five invasive and five non-invasive) and five transplants (two invasive and three non-invasive) and two cell lines (NI-11 and NI-12). Of these samples, nine primary tumours and three transplants were also used in DNA transfection assay. No amplification was demonstrated in any sample including NI-10, which contained activated H-*ras* gene.

### *Detection of activated* ras *p21 by immunoblot*

All of the 18 tumour samples tested demonstrated p21 at various densities (Figure 3). Seven of 18 carcinomas contained activated *ras* p21 reactive with monoclonal antibody E184 which specifically recognises the mutation from glycine to glutamic acid at codon 12. The protein extract of NI-10 which was shown to contain activated H-*ras* gene by Southern blot analysis also expressed activated *ras* p21. No reactivity was demonstrated with monoclonal antibodies DWP and R256 or negative controls MOPC-21 and 141 (data not shown).

### Discussion

In the present study, we not only confirmed the previous observation that the frequency of deeply invasive carcinomas was proportional to MNU dose, but that the highest dose schedule (0.5 mg × 12) was able to shorten the induction period considerably. The tumour implants in nude mice remained relatively stable in their phenotypic expression after repeated passages.

One mechanism of activation of *ras* genes is induction of mutation at positions 12, 13 or 61 (Tabin *et al*., 1982; Reddy *et al*., 1982; Bos *et al*., 1985; Yuasa *et al*., 1983). In MNU-induced rat mammary carcinomas, the H-*ras*-1 became activated by single amino acid substitution at the twelfth codon, encoding glutamic acid instead of glycine (Sukumar *et al*., 1983). To clarify the effect of MNU on the *ras* p21 in MNU-induced rat bladder carcinomas, we examined the reactivity of *ras* p21 with antibodies which were raised against synthetic peptides showing substitution at codon 12 of *ras* p21 from glycine to valine, glutamic acid, or arginine. Specificity of these monoclonal antibodies has been adequately demonstrated (Carney *et al*., 1986; Pullano *et al*., 1989; Azuma *et al*., 1990). Immunoblot analysis demonstrated that seven of 18 carcinomas tested contained activated *ras* p21 with substitution with glutamic acid. Of the nine tumours which were subjected also to DNA transfection assay, three showed twelfth codon mutation and yet in only one of these (NI-10) *ras* gene activation was demonstrable by Southern blot hybridisation perhaps due to low sensitivity of the assay. It has been observed that although human H-*ras*-1 genes mutated at codon 12, encoding glutamic acid in place of glycine, generated transformants by transfection assay, the cells displayed a less striking change in morphology as compared to those generated by mutated *ras* genes which encoded valine (Seeburg *et al*., 1984). Since rare 'spontaneous' carcinoma occurred in urine-treated HTB without carcinogen treatment (Ozono *et al*., 1983) there is a possibility that *ras* mutation seen in some tumours is unrelated to MNU treatment.

In conclusion, our data indicate that approximately one-half of non-invasive and invasive carcinomas induced by MNU contain activated *ras* oncogenes or oncogene product p21, but that their expression cannot be correlated to the aggressiveness of tumours. Our data are consistent with the previous observation that H-*ras* oncogenes are activated by MNU during the initiation of rat mammary carcinogenesis (Sukumar *et al*., 1983).

We are grateful to Dr Mariano Barbacid, Squibb Institute For Medical Research, Princeton, NJ, for providing us with S2-721 and 118-413 cells.

This investigation was supported by NIH grant CA 14649.

### References

ANDERSSON, P., GOLDFARB, M.M. & WEINBERG, R.A. (1979). A defined subgenomic fragment of *in vitro* synthesized Molony sarcoma virus DNA can induce cell transformation upon transfection. *Cell*, **16**, 63.

AZUMA, M., MOMOSE, H. & OYASU, R. (1990). *In vitro* malignant conversion of low-grade rat urinary bladder carcinoma cells by exposure to N-methyl-N-nitrosourea. *Cancer Res*., **50**, 7062.

BABAYA, K., MIYATA, Y., CHMIEL, J.S. & OYASU, R. (1982). Effects of rat urine fractionated by molecular weight on urinary bladder carcinogenesis. *Cancer Res*., **42**, 15.

BOS, J.L., TOKSOZ, D., MARSHALL, C.J. & 6 others (1985). Amino-acid substitutions at codon 13 of the N-*ras* oncogene in human acute myeloid leukemia. *Nature*, **315**, 726.

BRAWN, P.N. (1982). The origin of invasive carcinoma of the bladder. *Cancer*, **50**, 515.

CARNEY, W.P., PEPIT, D., HAMER, P. & 10 others (1986). Monoclonal antibody specific for an activated *ras* protein. *Proc. Natl Acad. Sci. USA*, **83**, 7485.

DEBIEC-RYCHTER, M., ZUKOWSKI, K. & WANG, C.Y. (1989). Chromosomal characteristics and malignancy of urothelial cells from carcinogen-treated rats. *J. Natl Cancer Inst*., **81**, 361.

FINKEL, T., DER, C.J. & COOPER, G.M. (1984). Activation of *ras* genes in human tumors does not affect localization, modification, or nucleotide binding properties of p21. *Cell*, **37**, 151.

FUJITA, J., OHUCHI, N., ITO, N. & 4 others (1988). Activation of H-*ras* oncogenes in rat bladder tumors induced by N-butyl-N-(4-hydroxybutyl)nitrosamine. *J. Natl Cancer. Inst*., **80**, 37.

FUJITA, J., SRIVASTAVA, S.K., KRAUS, M.H., RHIM, J.S., TRONICK, S.R. & AARONSON, S.A. (1985). Frequency of molecular alteration affecting *ras* proto-oncogenes in human urinary tract tumors. *Proc. Natl Acad. Sci. USA*, **82**, 3849.

FUJITA, J., YOSHIDA, O., YUASA, Y., RHIM, J.S., HATANAKA, M. & AARONSON, S.A. (1984). H-*ras* oncogenes are activated by somatic alterations in human urinary tract tumors. *Nature*, **309**, 464.

FURTH, M.E., DAVIS, L.J., FLEURDELYS, B. & SCOLNICK, E.M. (1982). Monoclonal antibodies to the p21 products of the transforming gene of Harvey murine sarcoma virus and of the cellular *ras* gene family. *J. Virol*., **43**, 294.

HIRAO, Y., IZUMI, K. & OYASU, R. (1980). The effect of normal rat urine on mucosal regeneration in heterotopic urinary bladder. *Lab. Invest*., **42**, 76.

KAYE,. K.W. & LANGE, P.H. (1982). Mode of presentation of invasive bladder cancer: reassessment of the problem. *J. Urol*., **128**, 31.

KUNZE, E. (1979). Development of urinary bladder cancer in the rat. In *Carcinogenesis: Current Topics in Pathology*, Grundmann, E. (ed.) p. 151, Springer-Verlag: Berlin.

LAEMMLI, U.K. (1970). Cleavage of structural proteins during the assembly of the head of bacteriophage T4. *Nature*, **227**, 680.

MEYERS, F.J., GUMERLOCK, P.H., KOKORIS, S.P., DE VERE WHITE, R.W. & MCCORMICK, F. (1989). Human bladder and colon carcinomas contain activated *ras* p21. *Cancer*, **63**, 2177.

OYASU, R., IWASAKI, T., MATSUMOTO, M., HIRAO, Y. & TABUCHI, Y. (1978). Induction of tumors in heterotopic bladder by topical application of N-methyl-N-nitrosourea and N-butyl-N-(3-carboxypropyl)nitrosamine. *Cancer Res*., **38**, 3019.

OYASU, R., MANNING, D.J., MATSUMOTO, M. & HOPP, M.L. (1976). Heterotopic urinary bladder with a communicating reservoir. *Cancer Res*., **36**, 2261.

OYASU, R., SAMMA, S., OZONO, S., BAUER, K., WALLEMARK, C.-B. & HOMMA, Y. (1987). Induction of high-grade, high-stage carcinomas in the rat urinary bladder. *Cancer*, **59**, 451.

OZONO, S., BABAYA, K., MORIKAWA, A. & OYASU, R. (1983). A minimal dose of N-methyl-N-nitrosourea carcinogenic to heterotopically transplanted rat urinary bladder. *Carcinogenesis*, **4**, 547.

PULLANO, T.G., SINN, E. & CARNEY, W.P. (1989). Characterization of monoclonal antibody R256, specific for activated *ras* p21 with arginine at 12, and analysis of breast carcinoma of v-Harvey-*ras* transgenic mouse. *Oncogene*, **4**, 1003.

REDDY, E.P., REYNOLDS, R.K. & SANTOS, E. (1982). A point mutation is responsible for the acquisition of transforming properties by the T24 human bladder carcinoma oncogene. *Nature*, **300**, 149.

RIGBY, P., DICKMAN, R., RHODES, C. & BERG, P. (1977). Labelling of deoxyribonucleic acid to high specific activity *in vitro* by nick translation with DNA polymerase. *J. Mol. Biol.*, **98**, 503.

SEEBURG, P.H., COLBY, W.W., CAPON, D.J., GOEDDEL, D.V. & LEVINSON, A.D. (1984). Biological properties of human c-Ha-*ras* 1 genes mutated at codon 12. *Nature*, **312**, 71.

SUKUMAR, S., NOTARIO, V., MARTIN-ZANCA, D. & BARBACID, M. (1983). Induction of mammary carcinomas in rats by nitrosomethylurea involves malignant activation of H-*ras*-1 locus by single point mutations. *Nature*, **306**, 658.

TABIN, C.J., BRADLEY, S.M., BARGMANN, C.I. & 6 others (1982). Mechanism of activation of a human oncogene. *Nature*, **300**, 143.

TOWBIN, H., STAEHLIN, T. & GORDON, J. (1979). Electrophoretic transfer of proteins from polyacrylamide gels to nitro-cellulose sheets: procedure and some applications. *Proc. Natl Acad. Sci. USA*, **76**, 4350.

YUASA, Y., SRIVASTAVA, S.K., DUNN, C.Y., RHIM, J.S., REDDY, E.P. & AARONSON, S.A. (1983). Acquisition of transforming properties by alternative point mutations within C-bas/has protooncogene. *Nature*, **303**, 775.